# Pulse Steroid Therapy for Severe Acute Respiratory Distress Syndrome: A Propensity Score-Matched Analysis

**DOI:** 10.3390/jcm14155547

**Published:** 2025-08-06

**Authors:** Yasumasa Kawano, Junichi Maruyama, Mitsuaki Nishikimi, Hisatomi Arima, Yuhei Irie, Shinichi Morimoto, Kentaro Muranishi, Maiko Nakashio, Yoshihiko Nakamura

**Affiliations:** 1 Department of Emergency and General Medicine, Fukuoka University Chikushi Hospital, Chikusino 818-8502, Japan; 2 Department of Emergency and Critical Care Medicine, Faculty of Medicine, Fukuoka University Hospital, Fukuoka 814-0180, Japan; marujun1025@icloud.com (J.M.); iriey8383@gmail.com (Y.I.); otomirom7960@yahoo.co.jp (S.M.); rabbit.19871227@gmail.com (K.M.); smapmaiko0428@gmail.com (M.N.); nakamura58@adm.fukuoka-u.ac.jp (Y.N.); 3 Department of Emergency and Critical Care Medicine, Graduate School of Biomedical and Health Sciences, Hiroshima University, Hiroshima 739-8511, Japan; nishikim@hiroshima-u.ac.jp; 4 Department of Preventive Medicine and Public Health, Faculty of Medicine, Fukuoka University, Fukuoka 814-0180, Japan; harima@fukuoka-u.ac.jp

**Keywords:** methylprednisolone, corticosteroid, respiratory failure, venovenous ECMO

## Abstract

**Background/Objectives:** Low-dose corticosteroids have gained popularity in the treatment of acute respiratory distress syndrome (ARDS); however, the efficacy of high-dose corticosteroids as pulse steroid therapy remains controversial. This study aimed to evaluate the efficacy of pulse steroid therapy in patients with severe ARDS requiring venovenous (V-V) extracorporeal membrane oxygenation (ECMO), where enhanced anti-inflammatory effects may be beneficial. **Methods:** Using data from the J-CARVE registry, which included patients with severe ARDS managed with V-V ECMO across 24 Japanese hospitals between January 2012 and December 2022, we identified 373 patients treated with corticosteroids. The patients were divided into two groups: pulse steroid therapy and non-pulse steroid therapy. Propensity score matching was performed, and all-cause hospital mortality and ECMO-free days within 28 days were compared between groups. Pulse steroid therapy was defined as methylprednisolone at a dose of 1000 mg/day. **Results:** After matching, 48 patients were included in each group. The all-cause hospital mortality rates were 41.7% (20/48) in the pulse steroid group and 47.9% (23/48) in the non-pulse steroid group, with no significant difference (odds ratio, 1.28; 95% confidence interval: 0.53–3.12, *p* = 0.68). The median ECMO-free days were 9.5 (interquartile range [IQR]: 0–17.3) in the pulse steroid group and 3 (IQR: 0–17) in the non-pulse steroid group, showing no significant difference (*p* = 0.69). **Conclusions**: Pulse steroid therapy did not improve all-cause hospital mortality or ECMO-free days in patients with severe ARDS who required V-V ECMO.

## 1. Introduction

Localized damage caused by pneumonia or systemic conditions (e.g., transfusion-associated acute lung injury, severe trauma, pancreatitis) frequently leads to acute respiratory failure, which is defined as acute respiratory distress syndrome (ARDS) if it meets the Berlin definition [1]. ARDS continues to have a high mortality rate despite advancements in adjunctive treatments, such as prone positioning and neuromuscular blockade [2], with mortality rates exceeding 50% in patients receiving venovenous (V-V) extracorporeal membrane oxygenation (ECMO) [3], which underscores the need for continued clinical focus on this syndrome.

In early ARDS, lung involvement is characterized by diffuse alveolar damage caused by proinflammatory mediators [4]. In contrast, corticosteroid administration reduces proinflammatory cytokine levels [5]. Therefore, corticosteroids, as potent anti-inflammatory agents, are expected to be effective in treating ARDS [6]; the beneficial effects of corticosteroids in patients with ARDS have been demonstrated in several studies [7,8]. For example, in one trial, patients treated with corticosteroids, compared to the placebo group, exhibited a significantly higher rate of extubation or improvement in lung injury scores by Day 7 [7]. Similarly, a larger cohort study found that patients receiving dexamethasone had more ventilator-free days at 28 days than those receiving placebo, with lower mortality rates in the dexamethasone group than in the placebo group at 60 days [8]. However, these studies did not focus on patients with the most severe forms of ARDS requiring V-V ECMO. To our knowledge, no previous study has exclusively examined the efficacy of corticosteroid therapy in this subgroup of critically ill patients. As such, the optimal corticosteroid regimen for patients requiring ECMO support remains unclear. Pulse-dose steroids involve the administration of very high doses of glucocorticoids, typically methylprednisolone, ranging from 10 to 20 mg/kg or >250 mg/day and as high as 1 g/day [9]. The rationale for using pulse-dose steroids is that these high doses may exert a strong anti-inflammatory effect, potentially counteracting the hyper-inflammatory phase of ARDS and improving patient outcomes. This approach may be particularly beneficial in patients with fulminant ARDS receiving ECMO, in whom conventional steroid doses may be insufficient to control inflammation. However, to date, no studies have investigated the efficacy of pulse steroid therapy in critically ill patients. Furthermore, the use of corticosteroids in ECMO-supported ARDS patients varies considerably across institutions, partly due to the lack of robust evidence specific to this subgroup. Therefore, this study aimed to examine the prognostic impact of pulse steroid therapy versus non-pulse steroid therapy in patients with acute respiratory failure requiring V-V ECMO.

## 2. Materials and Methods

### 2.1. Setting

This retrospective observational study utilized the Japan Chest computed tomography (CT) for ARDS requiring V-V ECMO registry (J-CARVE registry), a database of patients with severe ARDS receiving V-V ECMO, which included data from 24 institutions across Japan. The J-CARVE registry aims to evaluate chest CT imaging data at the initiation of V-V ECMO support between January 2012 and December 2022 [10]. The included patients were those aged ≥18 years who had been admitted to the study ICUs between January 2012 and December 2022 for the treatment of severe ARDS with V-V ECMO support, diagnosed based on the Berlin definition criteria (PaO_2_/FiO_2_ ratio ≤ 100) [1]. The participating institutions were required to have treated at least 10 patients with severe ARDS requiring V-V ECMO during the study period, ensuring sufficient experience with this treatment modality. This post hoc study was approved by the Institutional Review Board of Fukuoka University Chikushi Hospital (number: C24-12-005).

### 2.2. Data Collection

The following information was collected from the J-CARVE registry dataset: age, sex, body mass index (BMI), causes of ARDS, total Sequential Organ Failure Assessment (SOFA) score [11], respiratory ECMO survival prediction (RESP) score [12], Murray lung injury score, number of days from intubation to ECMO initiation, prone positioning and neuromuscular blockers use before and after initiation of ECMO, comorbidities, laboratory tests (white blood cell count, platelet count, C-reactive protein, D-dimer) at the time of admission, arterial blood gas tests before ECMO initiation, type and dosage of steroid therapy administered during ECMO management and within two weeks of initiating ECMO treatment, and renal replacement therapy. Additionally, data on all-cause hospital mortality, ECMO therapy duration, and adverse events were collected to evaluate the endpoints.

### 2.3. Patient Selection

Patients who did not receive steroids were excluded. Furthermore, patients with the following missing data were excluded: laboratory tests, SOFA score, Murray lung injury score, RESP score, BMI, steroid use, adverse events, prone therapy, and duration of ECMO treatment. We defined pulse steroid treatment as ≥1 g of methylprednisolone/day in terms of potency, and eligible patients were then allocated to either the pulse or non-pulse steroid groups.

### 2.4. Endpoints

The primary endpoint of this study was all-cause hospital mortality, while the secondary endpoints included ECMO-free days (ECMOFDs) on day 28 and adverse events, with the latter assessed using the rate of positive blood cultures as an indicator of infectious events and the rate of intracranial bleeding as an indicator of bleeding events. ECMOFDs were calculated as follows: ECMOFDs = 0 if the patient died within 28 days; and ECMOFDs = (28 − x) if ECMO management was terminated within 28 days, wherein x is the number of days of ECMO treatment.

### 2.5. Statistical Analysis

The results are expressed as median (interquartile range [IQR]) for continuous data and as percentages for categorical data. One-to-one nearest-neighbor matching without replacement was performed between the pulsed and non-pulsed groups based on the estimated propensity scores for each patient. To estimate the propensity score, a logistic regression model was fitted for patients who underwent pulse treatment as a function of age, sex, BMI, causes of ARDS, prone therapy before and after ECMO initiation, underlying collagen disease, initiation of ECMO within 7 days of tracheal intubation, total SOFA score, RESP score, Murray lung injury score, laboratory tests (white blood cell count and C-reactive protein level), and arterial blood gas tests before ECMO initiation (pH and PaCO_2_). A caliper width equal to 0.2 of the standard deviation of the propensity score logit was used. The standardized difference was used to evaluate the covariate balance. After matching, 48 pairs (96 patients) were included in the final analysis. The C-statistic for the propensity score model was 0.837, indicating good model discrimination. To evaluate the differences between the pulse and non-pulse steroid groups, categorical variables were compared using Fisher’s exact test, whereas continuous variables were compared using the Mann–Whitney U test. Forest plots were generated to visualize the association between pulse steroid therapy and all-cause hospital mortality, stratified by subgroups including underlying collagen disease or interstitial pneumonia, ECMO initiation within 7 days of intubation, and age and sex. Differences in the effects of pulse steroid therapy between the subgroups were tested by adding interaction terms to the statistical models. All tests were two-tailed, and a *p*-value of <0.05 was considered statistically significant.

All statistical analyses were performed using EZR software (version 1.68, Saitama Medical Center, Jichi Medical University, Saitama, Japan) [13], which is a graphical user interface for R software (version 4.20, The R Foundation for Statistical Computing, Vienna, Austria). More precisely, it is a modified version of the R commander, which is designed to add statistical functions frequently used in biostatistics.

## 3. Results

### 3.1. Patients

**Figure 1:**
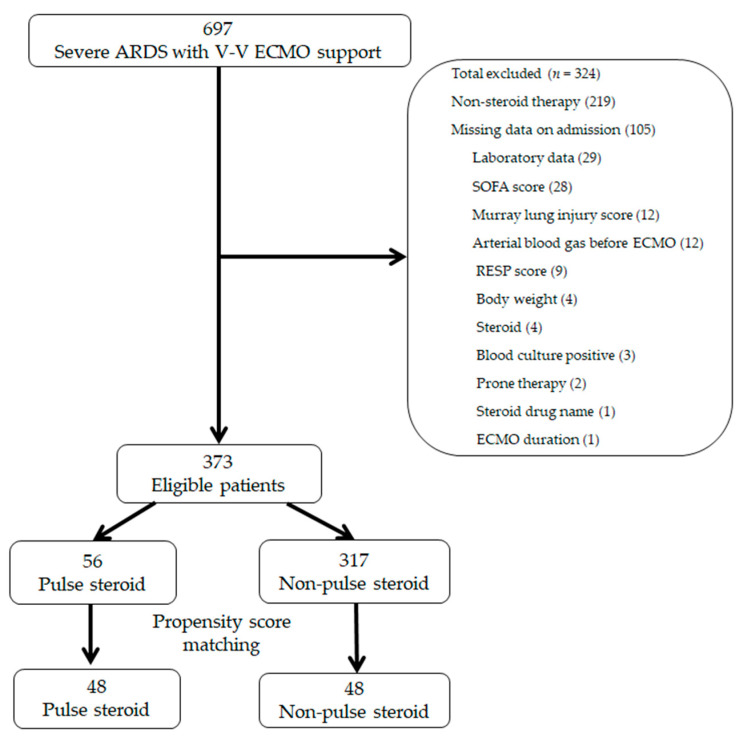
Patient selection schema. ARDS, acute respiratory distress syndrome; ECMO, extracorporeal membrane oxygenation; SOFA, Sequential Organ Failure Assessment; RESP, respiratory extracorporeal membrane oxygenation survival prediction.

This study enrolled 697 patients during the observation period; we excluded 219 patients who did not receive steroid therapy and 105 patients for whom the required data were unavailable. Finally, 373 eligible patients were categorized into the pulse (*n* = 56) or non-pulse (control group; *n* = 317) steroid groups, from which 48 propensity score-matched pairs were generated (Figure 1). The C-statistic indicated that the goodness of fit was 0.837 in the propensity score model.

The baseline characteristics of the unmatched pulse and non-pulse steroid groups and those of the propensity score-matched groups are shown in Table 1 and Table 2. When the unmatched groups were compared, BMI, SOFA score, prone therapy before and after ECMO initiation, underlying collagen disease, underlying interstitial pneumonia, underlying cirrhosis, and D-dimer levels differed significantly between the two groups. All patients in the pulse steroid group received methylprednisolone. After matching the propensity score, the classifications of the causes of ARDS were bacterial in 24% (23/96), COVID-19 in 20.8% (20/96), and unknown in 41.7% (40/96) of the patients. In addition, the details of steroid therapy for patients classified in the non-pulse group are presented in Table 3.

### 3.2. Endpoints

In total, 23 of 56 patients (41.1%) who received pulse steroid therapy died in the hospital, compared with 109 of 317 (34.4%) patients who did not receive pulse therapy (odds ratio: 0.75, 95% confidence interval 0.41–1.41, *p* = 0.36). After propensity score matching, no significant difference was found in all-cause hospital mortality between the two groups (pulse vs. non-pulse: 41.7% vs. 47.9%, respectively; odds ratio: 1.28; 95% confidence interval: 0.53–3.12; *p* = 0.68). Additionally, in the propensity score-matched groups, the number of ECMOFDs within the first 28 days did not differ significantly between the two groups (9.5 [IQR: 0–17.3] vs. 3 [IQR: 0–17] days, respectively; *p* = 0.69). Similarly, no significant differences in adverse events between the two groups were found after propensity score matching (all *p* > 0.05; Table 4).

**Figure 2:**
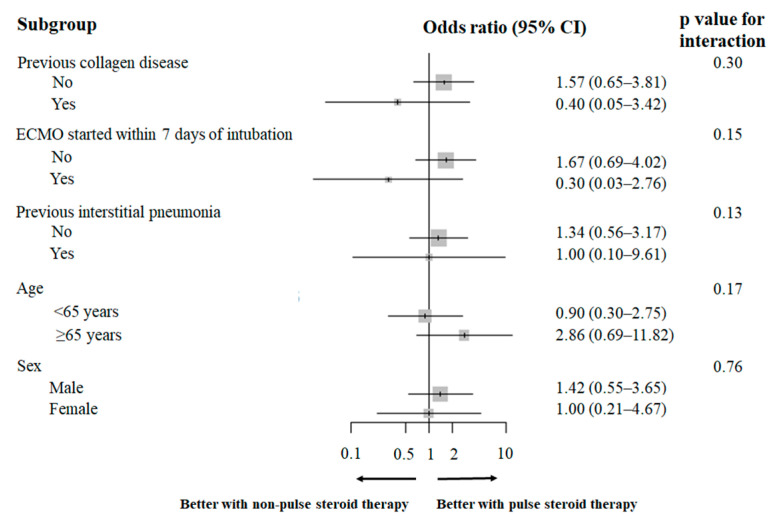
Forest plot of odds ratios for the association between pulse steroid therapy and all-cause hospital mortality by subgroups. CI, confidence interval; ECMO, extracorporeal membrane oxygenation.

Forest plots showed no significant differences in the association between pulse steroid treatment and all-cause in-hospital mortality across any subgroup (Figure 2).

## 4. Discussion

Compared to previous studies, this study included the largest number of patients with severe ARDS who underwent V-V ECMO; in addition, the patients were treated at 24 Japanese institutions. Our data did not indicate superiority of the pulse steroid group over the non-pulse steroid group in terms of in-hospital mortality and 28 ECMOFDs. No comparative study of pulse steroid and non-pulse steroid groups has been limited to patients with the most severe respiratory failure requiring V-V ECMO, and these data are noteworthy. As various baseline characteristics differed between the pulse (*n* = 56) and non-pulse (*n* = 317) steroid groups, comparison of all data for these two regimens was not valid. Therefore, to adjust for differing baseline characteristics, we extracted 48 comparable participants from each group by propensity score matching. As a result, the baseline characteristics were as homogenized as possible between the groups; therefore, the most valid conclusions could be drawn by comparing the pulse steroid group (*n* = 48) with the non-pulse steroid group (*n* = 48).

The patients with ARDS in this study included a heterogeneous group with regard to steroid sensitivity. Previous reports have indicated that steroids may be effective in ARDS caused by pneumocystis pneumonia [14], idiopathic organizing pneumonia [15], eosinophilic pneumonia [16], interstitial pneumonia related to autoimmune diseases [17], and COVID-19 [18]. However, previous research suggests that steroids may be less effective against influenza pneumonia [19] and community-acquired pneumonia [20] and are completely ineffective against carcinomatous lymphangitis [21] and alveolar proteinosis [22]. In this study, the cause of ARDS was not diagnosed in 41.7% of the cases, and many of these patients may not have benefited from steroids, which may have contributed to the lack of efficacy of pulse steroid therapy.

Although hospital mortality did not differ remarkably between the pulse and non-pulse groups, this finding should be interpreted with caution. In populations with a low proportion of unknown ARDS etiologies or a high prevalence of steroid-responsive conditions, outcomes may vary, as these characteristics can influence both corticosteroid response and overall prognosis.

Additionally, steroid adsorption by the ECMO circuit may have reduced systemic exposure despite their administration. ECMO circuits can adsorb various medications [23], particularly those with high protein-binding properties [24], and corticosteroids are known to be highly protein-bound [25]. Although direct measurement of steroid loss via ECMO circuits has not been demonstrated, this mechanism remains theoretically plausible and may have contributed to the limited efficacy of pulse steroid therapy observed in this study.

Another possible explanation for the limited effectiveness of pulse steroid therapy is that potential benefits may have been offset by steroid—[26] or ECMO-related complications [27]. Although we were unable to examine all potential complications associated with steroids and ECMO, this seems unlikely, given that no significant difference was found in the incidence of intracranial hemorrhage or positive blood cultures between the pulse steroid and non-pulse steroid groups.

This study had some limitations. First, it was a retrospective study. Second, only the type and dosage of steroid therapy were examined; the optimal duration of steroid therapy was not investigated. Third, although all the participating hospitals adhered to the guidelines for V-V ECMO indications, the final decision was left to the discretion of each hospital. Fourth, no standardized protocol was applied regarding the timing, type, dosage, and duration of steroid administration, ARDS treatment, or V-V ECMO weaning, and each institution made its own judgments. Fifth, lung transplantation is rarely performed in Japan, and no cases of lung transplantation were present, which may have influenced decisions regarding the indication and discontinuation of V-V ECMO treatment. Sixth, we could not account for time-dependent biases in treatment initiation or changes in clinical status, nor could we assess the continuous, time-adjusted dose–response relationship of corticosteroids. Finally, because our cohort comprised patients with diverse ARDS etiologies, the efficacy of pulse-dose steroid therapy may vary across disease entities. Therefore, prospective studies, including randomized controlled trials in more homogeneous patient subgroups, are needed to clarify the role of high-dose corticosteroids in patients with severe ARDS requiring V-V ECMO.

## 5. Conclusions

Compared with non-pulse steroid requiring V-V ECMO, pulse steroid therapy did not improve all-cause hospital mortality in critically ill patients compared to non-pulse steroid therapy.

## Figures and Tables

**Table 1:** Patient characteristics according to study group.

Variables	Unmatched Group					Matched Group				
	All Patients (*n* = 373)	Non-Pulse (*n* = 317)	Pulse (*n* = 56)	SD	*p* Value	All Patients (*n* = 96)	Non-Pulse (*n* = 48)	Pulse (*n* = 48)	SD	*p* Value
Demographic factors and severity of illness										
Age, median (IQR), years	60 (51–67)	60 (51–67)	62.5 (52–67)	0.06	0.711	62 (48.8–69.3)	57 (45.8–70.3)	63 (53.8–67.0)	0.134	0.610
male sex, *n* (%)	283 (75.9)	245 (77.3)	38 (67.9)	0.213	0.131	70 (72.9)	35 (72.9)	35 (72.9)	<0.001	1.000
BMI, median (IQR)	26 (22.8–30.5)	26.2 (23.2–31.1)	24.2 (21.5–27.0)	0.461	0.002	24.6 (22.2–28.0)	25.1 (21.4–28.3)	24.6 (22.4–27.0)	0.134	0.674
Causes of ARDS, *n* (%)				1.243	-				0.368	0.871
COVID-19	217 (58.2)	207 (65.3)	10 (17.9)			20 (20.8)	10 (20.8)	10 (20.8)		
Bacterial	45 (12)	31 (9.8)	14 (25)			23 (24)	11 (22.9)	12 (25.0)		
Extra-pulmonary	20 (5.4)	18 (5.7)	2 (3.6)			6 (6.3)	4 (8.3)	2 (4.2)		
Influenza	10 (2.7)	8 (2.5)	2 (3.6)			3 (3.1)	1 (2.1)	2 (4.2)		
Legionnaires	5 (1.3)	5 (1.6)	0 (0)			0 (0.0)	0 (0)	0 (0)		
Aspiration pneumonitis	4 (1)	3 (0.9)	1 (1.8)			2 (2.1)	1 (2.1)	1 (2.1)		
Trauma	4 (1)	3 (0.9)	1 (1.8)			2 (2.1)	2 (4.2)	0 (0.0)		
Drowning	1 (4)	1 (0.3)	0 (0)			0 (0.0)	0 (0)	0 (0.0)		
Unknown	67 (18)	41 (12.9)	26 (46.4)			40 (41.7)	19 (39.6)	21 (43.8)		
SOFA score, median (IQR)	10 (8–12)	9 (7–12)	11 (8–12)	0.273	0.042	11 (8–13)	11 (8–13)	11 (8–12)	0.1	0.737
RESP score, median (IQR)	3 (1–4)	3 (1–4)	2 (0–4)	0.111	0.300	2 (0–4)	2 (0–4)	2 (0–4)	0.22	0.408
Murray lung injury score, median (IQR)	13 (11–14)	13 (11–14)	13 (11–14)	0.023	0.888	13 (11–14)	13 (11–14)	13 (11–14)	0.047	0.673
Intubation to ECMO start <7 days, *n* (%)	310 (83.1)	262 (82.6)	48 (85.7)	0.084	0.700	82 (85.4)	42 (87.5)	40 (83.3)	0.118	0.770
Neuromuscular blockers use before ECMO start, *n* (%)	177 (47.5)	153 (48.3)	24 (42.9)	0.109	0.472	39 (40.6)	21 (43.8)	18 (37.5)	0.128	0.678
Prone therapy before ECMO start, *n* (%)	83 (22.3)	79 (24.9)	4 (7.1)	0.499	0.003	7 (7.3)	3 (6.2)	4 (8.3)	0.08	1.000
Comorbidities, *n* (%)										
Diabetes mellitus	106 (28.4)	94 (29.7)	12 (21.4)	0.189	0.261	19 (19.8)	8 (16.7)	11 (22.9)	0.157	0.609
Chronic kidney disease	32 (8.6)	27 (8.5)	5 (8.9)	0.015	1.000	7 (7.3)	3 (6.2)	4 (8.3)	0.08	1.000
Cerebrovascular accident	32 (8.6)	25 (7.9)	5 (8.9)	0.038	0.790	10 (10.4)	5 (10.4)	5 (10.4)	<0.001	1.000
Asthma	27 (7.2)	22 (6.9)	5 (8.9)	0.074	0.578	8 (8.3)	5 (10.4)	3 (6.2)	0.151	0.714
COPD	24 (6.4)	20 (6.3)	4 (7.1)	0.033	0.770	5 (5.2)	1 (2.1)	4 (8.3)	0.284	0.362
Collagen disease	29 (7.8)	19 (6.0)	10 (17.9)	0.372	0.005	15 (15.6)	8 (16.7)	7 (14.6)	0.057	1.000
Ischemic heart disease	21 (5.6)	18 (5.7)	3 (5.4)	0.014	1.000	5 (5.2)	2 (4.2)	3 (6.2)	0.09	1.000
Interstitial pneumonia	22 (5.9)	15 (4.7)	7 (12.5)	0.28	0.032	12 (12.5)	6 (12.5)	6 (12.5)	<0.001	1.000
Chronic heart failure	15 (4)	12 (3.8)	3 (5.4)	0.075	0.480	3 (3.1)	0 (0)	3 (6.2)	0.365	0.242
Lung cancer	8 (2.1)	7 (2.2)	1 (1.8)	0.03	1.000	3 (3.1)	2 (4.2)	1 (2.1)	0.12	1.000
Cirrhosis	7 (1.9)	3 (0.9)	4 (7.1)	0.319	0.011	4 (4.2)	0 (0)	4 (8.3)	0.426	0.117

ARDS, acute respiratory distress syndrome; BMI, body mass index; COVID-19, coronavirus disease 2019; COPD, chronic obstructive pulmonary disease; ECMO, extracorporeal membrane oxygenation; IQR, interquartile range; RESP, respiratory extracorporeal membrane oxygenation survival prediction; SOFA, Sequential Organ Failure Assessment; SD, standardized difference.

**Table 2:** Characteristics of patients, laboratory findings, and treatment for ARDS according to study group.

Variables	Unmatched Group					Matched Group				
	All Patients (*n* = 373)	Non-Pulse (*n* = 317)	Pulse (*n* = 56)	SD	*p* Value	All Patients (*n* = 96)	Non-Pulse (*n* = 48)	Pulse (*n* = 48)	SD	*p* Value
Laboratory data at ICU admission, median (IQR)										
WBC, 109/L	11.8 (8.3–17)	11.7 (8.2–16.1)	12.5 (9.5–19.8)	0.221	0.111	13.6 (10.4–20.2)	14.9 (11.5–20.8)	12.5 (10.3–20.0)	0.093	0.450
Platelet count, 109/L	190 (121–264)	189 (126–260)	192 (104–284)	0.117	0.787	190 (105–281)	161 (102–251)	198 (123–288)	0.22	0.206
CRP, mg/dL	10.6 (4.3–19)	10.4 (4.2–18.4)	12.4 (4.8–21.1)	0.185	0.233	12.2 (6.0–20.6)	10.9 (6.1–20.2)	12.5 (5.6–20.7)	0.022	0.826
D-dimer, μg/mL	5.9 (2.5–16)	5.5 (2.3–15.3)	9.6 (4.3–32.1)	0.115	0.028	8.5 (3.8–30.1)	7.9 (3.4–26.1)	9.4 (4.2–33.8)	0.17	0.575
Arterial gas data before starting ECMO										
pH	7.32 (7.22–7.39)	7.33 (7.23–7.39)	7.3 (7.2–7.4)	0.017	0.777	7.32 (7.21–7.39)	7.32 (7.23–7.38)	7.31 (7.21–7.39)	0.061	0.834
PaO_2_, mmHg	72.9 (60–89.6)	72.9 (60–87.5)	73 (59.5–90.4)	0.029	0.954	69.8 (59.0–91.1)	70.4 (58.7–93.3)	69.6 (60.0–89.8)	0.022	0.783
PaCO_2_, mmHg	50 (41–64.5)	50 (41–63)	49.2 (41–64.6)	0.084	0.887	49.4 (41.0–64.6)	49.0 (41.0–62.4)	50.1 (40.8–65.1)	0.163	0.703
HCO_3_^−^, mmol/L	26 (22.1–29)	26 (22–29)	26 (22.8–30.6)	0.093	0.528	25.7 (22.4–30.2)	25.0 (21.3–29.0)	26.0 (22.7–31.0)	0.205	0.234
Other therapeutic intervention, n (%)										
Steroid therapy				3.053	-				2.944	<0.001
Dexamethasone	173 (46.4)	173 (54.6)	0 (0)			13 (13.5)	13 (27.1)	0 (0)		
Methylprednisolone	112 (30)	56 (17.7)	56 (100)			57 (59.4)	9 (18.8)	48 (100)		
Hydrocortisone	31 (8.3)	41 (12.9)	0 (0)			13 (13.5)	13 (27.1)	0 (0)		
Prednisolone	37 (9.9)	37 (11.7)	0 (0)			12 (12.5)	12 (25.0)	0 (0)		
Betamethasone	10 (2.7)	10 (3.2)	0 (0)			1 (1.0)	1 (2.1)	0 (0)		
RRT	139 (37.3)	118 (37.2)	21 (37.5)	0.006	1.000	40 (41.7)	24 (50.0)	16 (33.3)	0.343	0.147
Prone therapy	137 (36.7)	127 (40.1)	10 (17.9)	0.505	0.001	18 (18.8)	11 (22.9)	7 (14.6)	0.215	0.433
Neuromuscular blockers use	237 (63.5)	206 (65)	31 (55.4)	0.198	0.178	58 (60.4)	32 (66.7)	26 (54.2)	0.258	0.297

ARDS, acute respiratory distress syndrome; CRP, C-reactive protein; ECMO, extracorporeal membrane oxygenation; ICU, intensive care unit; IQR, interquartile range; RRT, renal replacement therapy; SD, standardized difference; WBC, white blood cell count.

**Table 3:** Steroid therapy details in the non-pulse group (*n* = 48) among propensity score-matched groups.

Drug Name	Number (%)	Dose (mg)
Hydrocortisone	13 (27.1)	
	8	200
	2	300
	1	100
	1	60
	1	44
Dexamethasone	13 (27.1)	
	7	6.6
	3	20
	2	7
	1	6
Prednisolone	12 (25.0)	
	3	80
	3	60
	2	30
	1	90
	1	70
	1	40
	1	20
Methylprednisolone	9 (18.8)	
	3	80
	2	40
	1	500
	1	160
	1	88
	1	50
Betamethasone	1 (2.0)	
	1	16

**Table 4:** Outcomes and adverse events in the propensity-matched group analyses.

Variable	Unmatched Group					Matched Group				
	Non-Pulse (*n* = 317)	Pulse (*n* = 56)	OR	95%CI	*p* Value	Non-Pulse (*n* = 48)	Pulse (*n* = 48)	OR	95%CI	*p* Value
All-cause hospital mortality, *n* (%)	109 (34.4)	23 (41.1)	0.75	0.41–1.41	0.36	23 (47.9)	20 (41.7)	1.28	0.53–3.12	0.68
ECMOFDs on 28 days, median (IQR), days	15 (0–20)	11 (0–18)			0.24	3 (0–17)	9.5 (0–17.3)			0.69
Adverse events										
Blood culture positive, *n* (%)	99 (31.2)	5 (8.9)	0.22	0.07–0.56	<0.001	11 (22.9)	5 (10.4)	0.39	0.09–1.37	0.17
Intracranial bleeding, *n* (%)	10 (3.2)	1 (1.8)	0.56	0.01–4.07	1	2 (4.2)	1 (2.1)	0.49	0.01–9.77	1

CI, confidence interval; ECMOFDs, extracorporeal membrane oxygenation-free days; OR, odds ratio; IQR, interquartile range.

## Data Availability

The data supporting the findings of this study are available from the corresponding author upon reasonable request. Requests will be reviewed to determine whether they are subject to confidentiality obligations.

## Abbreviations

The following abbreviations are used in this manuscript:

ARDS	acute respiratory distress syndrome
BMI	body mass index
COVID-19	coronavirus disease 2019
COPD	chronic obstructive pulmonary disease
ECMO	extracorporeal membrane oxygenation
ECMOFDs	extracorporeal membrane oxygenation-free days
IQR	interquartile range
RESP	respiratory extracorporeal membrane oxygenation survival prediction
SOFA	Sequential Organ Failure Assessment
SD	standardized difference
